# *In Vitro* Pharmacological Screening of Essential Oils from *Baccharis parvidentata* and *Lippia origanoides* Growing in Brazil

**DOI:** 10.3390/molecules27061926

**Published:** 2022-03-16

**Authors:** Wilmer H. Perera, Alexander M. Scherbakov, Galina I. Buravchenko, Ekaterina I. Mikhaevich, Suzana Guimarães Leitão, Paul Cos, Andrey E. Shchekotikhin, Lianet Monzote, William N. Setzer

**Affiliations:** 1 CAMAG Scientific, Inc., 515 Cornelius Harnett Drive, Wilmington, NC 28401, USA; wilmer.perera@gmail.com; 2 Department of Experimental Tumor Biology, Blokhin N.N. National Medical Research Center of Oncology, 24 Kashirskoye sh., 115522 Moscow, Russia; a.sherbakov@ronc.ru (A.M.S.); k.mihaevich@gmail.com (E.I.M.); 3 Laboratory of Chemical Transformations of Antibiotics, Gause Institute of New Antibiotics, 11 B. Pirogovskaya St., 119021 Moscow, Russia; buravchenko.g.i@muctr.ru (G.I.B.); shchekotikhin@mail.ru (A.E.S.); 4 Organic Chemistry Department, Faculty of Natural Sciences, Mendeleyev University of Chemical Technology, 9 Miusskaya Square, 125190 Moscow, Russia; 5 Faculty of Pharmacy, Federal University of Rio de Janeiro, CCS, Bl. A, Ilha do Fundão, Rio de Janeiro 21941-902, Brazil; sgleitao@pharma.ufrj.br; 6 Research Network Natural Products against Neglected Diseases (ResNetNPND), University of Münster, 48149 Münster, Germany; paul.cos@uantwerpen.be (P.C.); monzote@ipk.sld.cu (L.M.); 7 Laboratory for Microbiology, Parasitology and Hygiene (LMPH), Faculty of Pharmaceutical, Biomedical and Veterinary Sciences, University of Antwerp, 2610 Antwerp, Belgium; 8 Parasitology Department, Institute of Tropical Medicine “Pedro Kouri”, Havana 10400, Cuba; 9 Department of Chemistry, University of Alabama in Huntsville, Huntsville, AL 35899, USA; 10 Aromatic Plant Research Center, 230 N 1200 E, Suite 100, Lehi, UT 84043, USA

**Keywords:** *Baccharis parvidentata*, *Lippia origanoides*, essential oil, protozoa, cancer cells

## Abstract

In this study, the *in vitro* antimicrobial, antiparasitic, antiproliferative and cytotoxic activities of essential oil from *Baccharis* *parvidentata* Malag. (EO-Bp) and *Lippia* *origanoides* Kunth (EO-Lo) were explored. The relevant effects were observed against the parasitic protozoans *Plasmodium falciparum*, *Trypanosoma cruzi*, *Trypanosoma brucei* and *Leishmania amazonensis* (ranging 0.6 to 39.7 µg/mL) and malignant MCF-7, MCF-7/HT, 22Rv1, and A431 cell lines (ranging 6.1 to 31.5 µg/mL). In parallel, EO-Bp showed better selective indexes in comparison with EO-Lo against peritoneal macrophages from BALB/c mice and MRC-5 cell line. In conclusion, EO-Lo is known to show a wide range of health benefits that could be added as another potential use of this oil with the current study. In the case of EO-Bp, the wide spectrum of its activities against protozoal parasites and malignant cells, as well as its selectivity in comparison with non-malignant cells, could suggest an interesting candidate for further tests as a new therapeutic alternative.

## 1. Introduction

The search for biologically active compounds is currently a subject of interest in the food, cosmetic and pharmaceutical industries, among others [1]. Plant-based natural products are a potential source of new agents and/or drugs [2]. The ability of plant species to biosynthesize a broad variety of complex and innovative scaffolds is outstanding and can be utilized or further developed as a major source of leads for new drugs if appropriate *in vitro* and *in vivo* assays are established. Numerous plant extracts and pure natural compounds have been discovered [3,4]. Among those, essential oils (EOs) have been shown to display a wide range of therapeutic properties [5,6]. EOs are a mixture of volatile compounds that have been found in different plant organs from several botanical families [5], including Asteraceae [7] and Verbenaceae [8]. In our previous study, the chemical and biological properties of the EOs from *Baccharis parvidentata* Malag. (Asteraceae) and *Lippia origanoides* Kunth (Verbenaceae) from high-altitude locations in Brazil were assessed [9].

Several of the curative properties found in the genus *Baccharis* have been ascribed to their EOs, which have been reported in the scientific literature to exhibit a wide range of biological properties [9,10,11]. However, the studies of *B. parvidentata* of the southeast of Minas Gerais and Rio de Janeiro States, Brazil, are scarce. The composition of the EOs from the aerial parts was recently reported, and sabinene (15.2%), himachalol (10.3%) and β-pinene (9.2%) were identified as the major volatile compounds [9].

On the other hand, the pharmacology of the genus *Lippia* has been widely examined. The content of EOs from the species of the genus *Lippia* is frequently variable [12,13] and exhibit numerous biological activities. The bioactivity of the EOs produced from *L. origanoides* has also been widely evaluated and exhibits numerous biological activities and has been summarized [14]. The EO composition of *L. origanoides* from Petropolis, Brazil, showed methyl (*E*)-cinnamate (40.0%), hedycaryol (8%), α-eudesmol (7.6%) and β-eudesmol (7.3%) as the most noteworthy compounds [9]. The high content of methyl (*E*)-cinnamate in the EO of *L. origanoides* herein evaluated, matched somewhat with the chemotype **E** previously described [14], which to the best of our knowledge has not been fully explored.

The therapeutic potential of the EOs has not been fully investigated; although a preliminary antimicrobial activity has been assayed that documented the minimal inhibitory concentrations (MIC) against some bacteria and fungi [9]. In this context, the purpose of this study was to explore *in vitro* the pharmacological properties of EOs from *B. parvidentata* (EO-Bp) and *L. origanoides* (EO-Lo), including antimicrobial, antiparasitic, antiproliferative, and cytotoxic activities.

## 2. Results and Discussion

The EOs were evaluated against a wide panel of microorganisms: Gram-negative *Escherichia coli*; Gram-positive *Staphylococcus aureus*; yeast *Candida albicans*; and protozoa *Plasmodium falciparum*, *Trypanosoma cruzi*, *T. brucei brucei*, *Leishmania amazonensis*, and *L. infantum*; malignant: MCF-7 (human breast cancer), MCF-7/HT (4-hydroxytamoxifen-resistant MCF-7/HT subline), 22Rv1 (human prostate carcinoma), and A431 (human epidermoid carcinoma); and non-malignant cells: MRC-5 (human fetal lung fibroblast), MCF-10A (normal breast cells), and peritoneal macrophage from BALB/c mice (PMM).

In general, the EOs showed low antimicrobial activity except for the EO-Bp that caused relevant growth inhibition of *S. aureus* with a median inhibitory concentration (IC_50_) value < 10 µg/mL (Table 1). Both EOs inhibited all protozoal parasites. EO-Bp displayed the best activity, *T. brucei* and *P. falciparum* were the most susceptible to its effect (Table 2). The protozoal parasites were also susceptible to EO-Lo, although with higher values of IC_50_, except for *L. amazonensis* equally susceptible to EO-Lo and EO-Bp and for *L. infantum,* which showed IC_50_ > 64 µg/mL. Moreover, both EOs showed antiproliferative activity (Table 3), but EO-Bp, with IC_50_ ranging from 6.1 to 15.1 µg/mL, was a better inhibitor of all malignant cell growth than EO-Lo with IC_50_ from 9.1 to 31.5 µg/mL.

**Figure 1:**
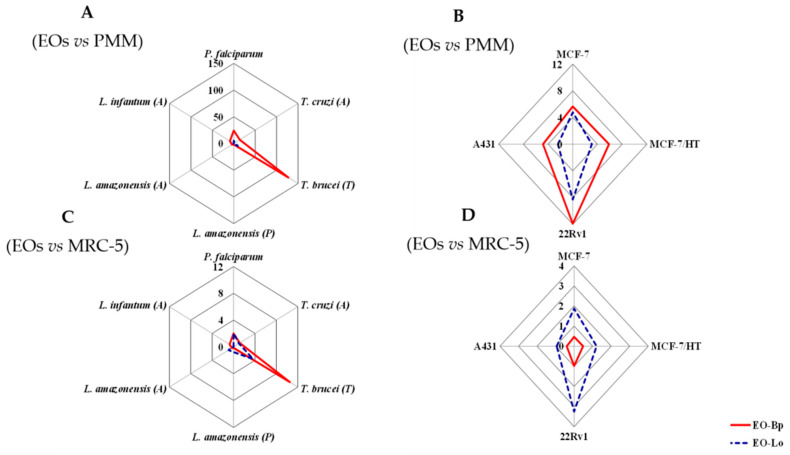
Selective indexes of essential oils from *Baccharis parvidentata* and *Lippia origanoides* from Brazil. (**A**) Antiprotozoal activity of EOs in comparison with cytotoxicity against PMM; (**B**) antiproliferative activity against malignant cells by EOs in comparison with cytotoxicity against PMM; (**C**) antiprotozoal activity of EOs in comparison with cytotoxicity against MRC-5; (**D**) antiproliferative activity against malignant cells of EOs in comparison with cytotoxicity against MRC-5. EOs: essential oils from *Baccharis parvidentata* and *Lippia origanoides*; EO-Bp: essential oil from *Baccharis parvidentata*; EO-Lo: essential oil from *Lippia origanoides*; PMM: peritoneal macrophage from BALB/c mice; MRC-5: human fetal lung fibroblast cells line.

In the same way, EO-Bp showed higher cytotoxic effects than EO-Lo in the used models (Table 3), according to the obtained median cytotoxic concentration (CC_50_). However, when the selectivity indices (SI) were obtained, the most promising activity was achieved for EO-Bp in PMM and MRC-5 models with respect to antiprotozoal activities (Figure 1A,C). In contrast, for the antiproliferative effect, EO-Bp showed better results in the PMM model, while for EO-Lo, better indexes were obtained with respect to the MRC-5 model (Figure 1B,D). In particular, with regards to the SI values calculated with respect to MCF-10A, both EOs showed unspecific activity in comparison with a malignant sensitive cell line (MCF-7) and a hormone-resistant cell subline (MCF-7/HT) with values around unity.

Brazil is reputed for its floristic diversity; hence its flora should be considered an important reservoir of active molecules with potential industrial applications [15]. Plant species from several botanical families have been described for their EO content and their antimicrobial [16], antiparasitic [17], anticancer [18], anti-oxidant [19], and insecticidal [20] activities. In the present study, the antiproliferative properties of EO from *B. parvidentata* and *L. origanoides*, collected in Brazil, against bacteria, fungi, protozoal parasites, malignant and non-malignant cells were investigated.

Although MIC values of these EOs: 625 to 1250 µg/mL against bacteria (*E. coli* and *S. aureus*), 156 to 2500 µg/mL against fungi (*Aspergillus niger*, *Fonsecaea pedrosoi*, and *Trycophyton rubrum*) and 78 to 2500 µg/mL against yeast (*C. albicans* and *Cryptococcus neoformans*) have been reported [9], our results confirm that no relevant activity was observed at 64 µg/mL, in both studied EOs except EO-Bp against *S. aureus*. It is known that *S. aureus* is a Gram-positive bacterium frequently found in the upper respiratory tract and on the skin as an opportunistic pathogen, being a common cause of sinusitis, abscesses, and food poisoning [21,22]. In this sense, EOs of other *Baccharis* species have been documented due to their potentialities against *S. aureus*, such as *B. dracunculifolia* DC [23] and *B. oreophila* Malme [24].

A wide inhibitory spectrum was appreciated against protozoal parasites of medical importance and malignant cells. Therefore, our screening strategy suggests the activity of the studied EOs against eukaryotic cells. The mechanisms underlying the antiprotozoal and antiproliferative actions of the tested EOs were not studied. Nevertheless, during the last decade, it has been postulated that the mechanism of action for oils and their constituents is rather complex. Several of these effects are attributable to the lipophilic nature and low molecular weight of the main components that comprise the EOs, which allow them to cross cell membranes, alter membrane composition, and increase membrane fluidity, leading to the leakage of ions and cytoplasmic molecules. Altering membranes leads to reduced ATP production, alteration of the pH gradient, and loss of mitochondrial potential, which can result in cell death. In addition, the induction of cell death by the activation of apoptotic and/or necrotic processes, cell cycle arrest, and loss of function of essential organelles has been observed. In parallel, some EOs may also act as pro-oxidant elements, which can alter the cellular redox state and compromise cellular survival. Nevertheless, the activities of EOs generally result from complex interactions between the different classes of compounds that can result in a great diversity of mechanisms of action and molecular targets [5].

The most sensitive parasite was *T. brucei*, which is responsible for African trypanosomiasis or sleeping sickness, mostly found in equatorial Africa. Human African trypanosomiasis takes two forms depending on the parasite involved, which are both transmitted by tsetse flies (*Glossina* spp.). Sleeping sickness in eastern and southern sub-Saharan Africa is an acute form caused by the subspecies *T. brucei rhodesiense*. Trypanosomiasis in the central and western regions of Africa is a slow-progressing form caused by *T. brucei gambiense*. Both trypanosomes invade the brain, causing mental deterioration, coma and death if left untreated [25,26].

The studied EOs displayed an important activity against 22Rv1 malignant cells, which are derived from human prostate carcinoma. In men, prostate cancer is the most commonly identified cancer and one of the most important causes of cancer-related deaths; inflammation is also associated with the pathogenesis of this disease [27,28]. The discovery and development of chemotherapeutic agents that can bind specifically prostate tumor cells is crucial to improve the treatment effectiveness and eventually avoid castration in affected patients [28,29].

Another point to consider is that EO-Bp showed the same IC_50_ value (*p* > 0.05) against susceptible MCF-7 and hormone-resistant MCF-7/HT (12.9 and 12.4 μg/mL, respectively). It is known that one of the hallmarks of cancer therapy resides in the ability of malignant cells to accumulate genetic/epigenetic changes until they achieve self-renewal, produce differentiated progeny, and develop resistance to therapy [30]. Thus, these results could represent a potential treatment opportunity for cancers that develop hormone resistance.

Moreover, toxicity on non-malignant cells is an important criterion in the drug development process. In that regard, EOs showed certain cytotoxicity, with CC_50_ values < 100 μg/mL. However, EO-Bp exhibited a selective antiparasitic effect on *T. brucei, T. cruzi* and *P. falciparum*, with SI values ranging from 14 to 128. Thus, these EOs should be considered for further exploration in animal models.

Another interesting result was that, in general, higher biological activities were observed for EO-Bp in comparison with EO-Lo (Table 1, Table 2 and Table 3), including: (i) number of susceptible organisms or cells: 7 vs. 5; (ii) average of all activities: 11.8 μg/mL vs. 22.5 μg/mL; (iii) range of activity against parasite: 0.6–39.7 μg/mL vs. 8.1–37.8 μg/mL, as well as malignant cell lines: 6.1–15.1 μg/mL vs. 9.1–31.5 μg/mL, and (iv) better SI with respect to parasites: 1–128 vs. 0–9 and malignant cells: 1–12 vs. 0–8, respectively. In addition, to the best of our knowledge, EO-Bp was analyzed in our study for the first time for its antiparasitic and antiproliferative potential; while EO-Lo was previously studied against protozoal parasites *T. evansi* [31], *T. cruzi* [32], and *L. chagasi* [33], and malignant cell line MDA-MB-231 [34]. Therefore, the higher potential activity of EO-Bp opens the possibility for further studies as a new therapeutic agent.

In Brazil, there are currently 167 species in the *Baccharis* genus; the country is one of the main centers of diversity of this genus [35,36]. The species of this genus are very relevant in folk medicine, with several species being used for the control and treatment of diseases, beyond the economic and environmental aspects. In this regard, a previous review highlights the antimicrobial and antiprotozoal activity of *Baccharis* species, which was considered a promising source of biologically active compounds [10]. In particular, the activity of EO-Bp have been scarcely explored; however, knowledge of the chemical structures may explain the observed biological activity and could serve as scaffolds for rational drug design, suggesting chemical modifications to increase activity, bioavailability, and toxicity, among other characteristics, allowing the design of new and more active compounds [17]. Then, to have a general overview of probable bioactive compounds of EO-Bp, reports retrieved from scientific literature related to the assayed pharmacological activities of identified major compounds are summarized in Table 4 [9]. In this sense, reports about the antimicrobial, antiparasitic and antiproliferative activities of the main compounds from EO-Bp were found. In some instances, although different cell targets in comparison with our study have been assayed, the versatility of activities is demonstrated and supports our results. Among these compounds, reports suggest that sabinene demonstrates a wide spectrum of antimicrobial and antiparasitic activities, while himachalol displayed antimicrobial and anticancer actions. In contrast, β-pinene was the most versatile compound and displayed antimicrobial, antiparasitic and anticancer activities.

## 3. Materials and Methods

### 3.1. Essential Oils

EO-Bp and EO-Lo were obtained previously by Perera et al. [9] and were obtained by common hydrodistillation in a Clevenger apparatus for 3 h and dried with anhydrous sodium sulfate. The EOs were chemically characterized using gas chromatography–mass spectrometry (GC–MS) and gas chromatography coupled to flame ionization detection (GC-FID) analyses (Supplemental Materials; Table S1). For biological assays, each EO was diluted in dimethyl sulfoxide (DMSO) at 20 mg/mL. This work is registered in SISGEN (Sistema Nacional de Gestão do Patrimonio Genetico, Brazil) under the access authorization number AC7DDF5.

### 3.2. Microorganisms and Cells

The reference strain of Gram-negative *E. coli* ATCC8739, Gram-positive *S. aureus* ATCC6538, yeast *C. albicans* B59630, and protozoa *P. falciparum* Ghana, *T cruzi* Tulahuen CL2, *T. brucei brucei* Squib-427, *L. amazonensis* MHOM/77BR/LTB0016, and *L. infantum* MHOM/MA(BE)/67 were used for assessing the EOs against infectious agents. In addition, four malignant cell lines were included: MCF-7 (ATCC^®^ HTB-22), MCF-7/HT (established in the laboratory), 22Rv1 (ATCC^®^ CRL-2505^TM^), and A431 (CRL-1555^TM^); three non-malignant cells were included MRC-5 (CCL-171™), MCF-10A (ATCC^®^ CRL-10317) breast cells, and PMM isolated at the moment of use from healthy animals (approved protocol number: CEI-IPK-68-20).

### 3.3. Antimicrobial Screening

The integrated antimicrobial screening in 96-well plates was adopted from Cos et al. [45]. Serial dilutions of the EOs were prepared from the DMSO stock solutions in sterile demineralized water in 96-well plates using an automated liquid-handling workstation (Beckman Coulter Biomek 3000), in which the final DMSO concentration was <1%. In all cases, negative (100% growing) and positive (a reference drug) control were included.

Antibacterial and antifungal assays were performed with 5 × 10^3^ CFU/well of *E. coli*, *S. aureus* (cultured in Mueller Hinton Broth medium (MHB) from Sigma-Aldrich, St. Louis, MO, USA) or *C. albicans* (cultured in RPMI medium from Sigma-Aldrich, St. Louis, MO, USA). EOs were added at concentrations ranging from 0.25 to 64 µg/mL. Then, plates were incubated 17 h at 37 °C, and viability was determined fluorimetrically by the addition of resazurin (Sigma-Aldrich, St. Louis, MO, USA) for 30 min at 37 °C or 4 h at 37 °C to bacteria and fungi cultures, respectively [46]. Finally, fluorescence was measured at 530 nm excitation and emission of 590 nm using a fluorimeter (Tecan Group, Maennedorf, Switzerland). Reference drugs were chloramphenicol (Sigma-Aldrich, Bornem, Belgium), erythromycin (Sigma-Aldrich, Bornem, Belgium) and miconazole (Janssen Pharmaceuticals, Beerse, Belgium) for *E. coli*, *S. aureus* and *C. albicans*, respectively.

The antiprotozoal assessment was carried out using different models, and parasite forms as will be described below. Activity against *P. falciparum* was measured with parasites cultured in human erythrocytes A+ (with 1% parasitemia and 2% hematocrit) in RPMI medium (with 0.5 (*g*/*v*)% AlbumaxTM) at 37 °C in an atmosphere of 3% O_2_, 4% CO_2_, and 93% N_2_ [47]. Suspensions of cells were distributed in a 96-well plate, treated with different concentrations of EOs and incubated for 72 h under the same conditions. Then, the plate was frozen at −20 °C, and parasite multiplication was measured after mixing 20 μL of the hemolyzed parasite suspension with 100 µL of MalstatTM (Flow Inc., Portland, OR, USA) reagent in a new plate. After 15 min of incubation at room temperature, 20 μL of nitro blue tetrazolium chloride (NBT; Sigma Aldrich, St. Louis, MO, USA), and 2 mg/mL/phenazine ethosulfate (Sigma Aldrich, St. Louis, MO, USA) solution was added. The plate was incubated again for 2 h at room temperature in the dark, and the absorbance was read in a Biorad 3550-UV microplate reader at 655 nm. Antitrypanosomal activity on amastigotes of *T. cruzi* was evaluated, using 4 × 10^4^ amastigotes in 4 × 10^3^ MRC-5 cells maintained in minimal essential medium (MEM; Life Technologies, Carlsbad, CA, USA) supplemented with 20 mM L-glutamine, 16.5 mM sodium bicarbonate and 5% of inactivated fetal calf serum. Then, EOs in tested concentrations were added and incubated for 7 days in the previous conditions. Parasite growth was assessed by adding the β-galactosidase substrate chlorophenol red β-D-galactopyranoside (Sigma Aldrich, St. Louis, MO, USA) subsequent to an additional incubation for 4 h at 37 °C. The absorbance was then read at 540 nm [48]. In the case of trypomastigotes of *T. brucei*, the assessment of activity was performed in Hirumi-9 medium supplemented with 10% inactivated fetal calf serum (FCSi) at 37 °C and 5% CO_2_ [49]. Assays were performed by adding 1.5 × 10^4^ trypomastigotes/well to the EOs at different concentrations and incubating them for 72 h under the same conditions. The fluorimetric resazurin method, with an additional incubation for 24 h at 37 °C, was used to measure parasite growth. Evaluation of activity against *L. amazonensis* promastigotes was carried out in a 96-well plate with 2 × 10^5^ parasites/mL and with the different concentrations of EOs. The plates were sealed with Parafilm and incubated at 26 °C for 72 h. Afterward, 20 μL of a solution (5 mg/mL) of MTT (3-[4,5-dimethylthiazol-2-yl]-2,5-diphenyltetrazolium bromide) (Sigma-Aldrich, St. Louis, MO, USA) was added to each well and incubated for an additional 4 h. The supernatant was removed, and the formazan crystals were dissolved with 100 μL DMSO. The absorbance of each well was determined with a plate reader (Molecular Devices, Sunnyvale, CA, USA) with a test wavelength of 560 nm and a reference wavelength of 630 nm [50]. On the other hand, the evaluation of antileishmanial activity against the intracellular amastigote form of *L. amazonensis* and *L. infantum* was also performed. In the model of *L. amazonensis*, a PMM monolayer was used (plated at 10^6^/mL in 24-well plates) and infected with stationary-phase promastigotes at a 4:1 parasite/macrophage ratio for 4 h at 5% CO_2_ and 37 °C. The cells were washed to remove free parasites. EOs were added at different concentrations, and the plates were further incubated under the same conditions for 48 h [51]. For *L. infantum*, 3 × 10^4^ PMM were infected with amastigotes obtained from an infected hamster at a ratio of 15 parasites per cell. The plate was incubated for 48 h at 37 °C and 5% CO_2_, and different concentrations of EOs were added. Then, the plate was incubated under the same conditions for 120 h. In both parasite species, the supernatant was discarded after the incubation period with the products; cells were fixed with methanol, stained with 10% Giemsa and microscopically examined (Motic, Japan) under immersion oil. Total parasite burden was determined by the number of infected macrophages and the number of amastigotes inside the macrophages. In the antiprotozoal assays, chloroquine, enznidazole, suramine and miltefosine (donated by Special Programme for Research and Training in Tropical Diseases from the World Health Organization (WHO-TDR)) were used as reference drugs for *P. falciparum, T. cruzi, T. brucei*, and *L. infantum*, respectively, while pentamidine (Richet, Buenos Aires, Argentina) was used for *L. amazonensis*.

### 3.4. Antiproliferative Assay on Malignant Cells

MCF-7, MCF-7/HT, 22Rv1 and A431 cancer cells were cultured in standard 4.5 g/L glucose DMEM medium (Gibco-Life Technologies, Paisley, UK), when 22Rv1 were cultivated in RPMI-1640 medium (Gibco-Life Technologies, Paisley, UK) supplemented with RPMI-1640 Vitamins (PanEco, Moscow, Russia). In all cases, the culture was supplemented with 10% fetal calf serum (FCS), antibiotics (50 μg of streptomycin/mL and 50 U of penicillin/mL) and 0.1 mg/mL sodium pyruvate (Santa Cruz Biotechnology, Dallas, TX, USA) and maintained in a NuAir incubator (NuAir, Plymouth, MN, USA) at 37 °C, 5% CO_2_ and 80–85% humidity. Then, 100 × 10^3^ 22Rv1 cells/well, 40 × 10^3^ MCF-7 or MCF-7/HT cells/well and 35 × 10^3^ A431 cells/well, were seeded into 24-well plates in 900 μL of the medium, and the plates were incubated for 24 h at 37 °C and 5% CO_2_. Subsequently, EOs or reference drug were added at different concentrations and the plates were incubated for 72 h under the same conditions. Cell viability was assessed using MTT at 0.2 mg/mL per well [52]. After additional incubation for 2 h, the supernatant was discarded, the MTT formazan purple crystals were dissolved in DMSO (350 μL per well) and the absorbance was measured at 571 nm and 630 nm as a reference in a MultiScan reader (ThermoFisher, Waltham, MA, USA) after the plates were gently shaken. Cisplatin (Teva Pharmaceutical Industries, Petah Tikva, Israel) was used as a reference drug.

### 3.5. Cytotoxicity Test on Non-Malignant Cells

The cytotoxicity analysis of EOs on 10^4^ MRC-5 cells/well was performed in MEM-supplemented medium at 37 °C and 5% CO_2_ seeded onto the test plates for 72 h. Cell viability was assessed fluorometrically after the addition of resazurin as described above. Cytotoxicity was also studied using MCF-10A cells cultured in medium supplemented with 5% donor horse serum (BioSera, Nuaille, France), 20 ng/mL epidermal growth factor (PanEco, Moscow, Russia), 0.5 µg/mL hydrocortisone (ChemCruz, Dallas, TX, USA), and 10 µg/mL insulin (PanEco) at 37 °C, 5% CO_2_ and 80–85% humidity). Briefly, 60 × 10^3^ MCF-10A cells were seeded into 24-well plates in 900 μL of the medium, and then, plates were incubated for 24 h at 37 °C and 5% CO_2_. Then, different concentrations of EOs were added, and the plates were incubated for 48 h under the same conditions. Cell viability was then measured by the MTT method as described above for cancer cell lines. Finally, in the model of PMM, 3 × 10^5^ cells/mL was treated with different concentrations of EOs over 48 h in the same conditions. Then, 15 μL of MTT solutions were added to each well, and, after 4 h of additional incubation in the same conditions, the supernatant was discarded, formazan crystals were dissolved with 100 μL of DMSO and the absorbance was obtained as described above.

### 3.6. Statistical Analysis

In each model included in this assessment, three experiments were performed and percentage growth inhibition for each product concentration was calculated compared to the untreated cultures (negative control). The IC_50_ for antibacterial, antifungal and antiprotozoal assays were determined, while CC_50_ was obtained in mammalian cell assays. In both cases, values were obtained from dose–response curves, and results were expressed as mean with a 95% confidence interval. Finally, SI was calculated as the ratio of the CC_50_ for MRC-5/PMM cells and the IC_50_ for microorganism or malignant cells.

## 4. Conclusions

In conclusion, EO-Lo is known to show a wide range of health benefits. The current study added another potential use of this oil in the area of infectious parasitic and malignant diseases. EO-Bp had antiparasitic and antiproliferative activity with a wide spectrum and selectivity in comparison with non-malignant eukaryotic cells. Thus, these products from a plant that grows in Brazilian regions can be considered interesting candidates for further tests as new therapeutic alternatives.

## Figures and Tables

**Table 1:** Antimicrobial and antiparasitic activity of essential oils from *Baccharis parvidentata* and *Lippia origanoides* growing in Brazil.

Infectious Agent	IC_50_ (μg/mL)		
	EO-Bp	EO-Lo	Rd
*E. coli*	>64	>64	0.8 (0.76–0.84)
*S. aureus*	9.2 (8.7–9.7)	>64	8.3 (7.9–8.7)
*C. albicans*	>64	>64	2.0 (1.9–2.1)
*P. falciparum*	3.0 (2.9–3.2)	14.4 (13.7–15.1)	0.02 (0.01–0.03)
*T. cruzi* (A)	5.2 (4.9–5.5)	29.6 (28.1–31.1)	0.8 (0.7–0.9)
*T. brucei* (T)	0.6 (0.5–0.7)	8.1 (7.7–8.5)	0.05 (0.02–0.08)
*L. amazonensis* (P)	39.7 (37.7–41.7)	37.8 (35.9–39.7)	0.4 (0.3–0.5)
*L. amazonensis* (A)	17.4 (16.5–18.3)	31.8 (30.2–33.4)	1.3 (1.2–1.4)
*L. infantum* (A)	8.1 (7.7–8.5)	>64	3.7 (3.5–3.9)

IC_50_: median inhibitory concentration with a 95% confidence interval. EO-Bp: essential oil from *Baccharis parvidentata.* EO-Lo: essential oil from *Lippia origanoides.* Rd: reference drug (chloramphenicol for *E. coli*; erythromycin for *S. aureus*; miconazol for *C. albicans*; chloroquine for *P. falciparum*; benznidazole for *T. cruzi*; suramine for *T. brucei*; pentamidine for *L. amazonensis*; miltefosine for *L. infantum*). Between the parentheses are parasite forms. A: amastigotes; T: trypomastigote; P: promastigotes.

**Table 2:** Antiproliferative activity of essential oils from *Baccharis parvidentata* and *Lippia origanoides* growing in Brazil.

Malignant Cell Line	IC_50_ (μg/mL)		
	EO-Bp	EO-Lo	Rd
MCF-7	12.9 (12.2–13.5)	15.8 (15.0–16.6)	1.9 (1.8–2.0)
MCF-7/HT	12.4 (11.8–13.0)	24.4 (23.2–25.6)	3.8 (3.6–4.1)
22Rv1	6.1 (5.8–6.4)	9.1 (8.6–9.5)	0.81 (0.77–0.85)
A431	15.1 (14.3–15.8)	31.5 (30.8–32.1)	1.2 (1.1–1.3)

IC_50_: median inhibitory concentration with a 95% confidence interval. EO-Bp: essential oil from *Baccharis parvidentata.* EO-Lo: essential oil from *Lippia origanoides.* Rd: reference drug (cisplatin).

**Table 3:** Cytotoxic *in vitro* effect of essential oils from *Baccharis parvidentata* and *Lippia origanoides* from Brazil.

Cell	CC_50_ (μg/mL)	
	EO-Bp	EO-Lo
PMM	72.8 (69.2–72.9)	75.5 (71.7–79.3)
MRC-5	6.0 (5.7–6.3)	29.6 (28.1–31.1)
MCF-10A	15.4 (13.9–16.9)	29.9 (27.1–32.7)

CC_50_: median cytotoxic concentration with a 95% confidence interval. EO-Bp: essential oil from *Baccharis parvidentata.* EO-Lo: essential oil from *Lippia origanoides.* PMM: peritoneal macrophage from BALB/c mice. MRC-5: human fetal lung fibroblast cells line. MCF-10A: normal breast cells line.

**Table 4:** In vitro pharmacological activities retrieved from scientific literature on major compounds identified in the essential oils from *Baccharis parvidentata* growing in Brazil.

Major Compounds	Pharmacological Property	Target (Results)	Reference
**Sabinene** 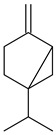	Antimicrobial	- Mycobacterium tuberculosis (MIC from 64 to >512 µg/mL)	[37]
		- *Salmonella typhimurium* (in silico study that demonstrated L-asparaginase as a drug target)	[38]
	Antiparasitic	- *Leishmania major* (IC_50_ = 126.6 µg/mL)	[39]
		- Trypanosoma brucei (IC_50_ = 17.7 µg/mL)	
	Antiproliferative	No reports were found	-
**Himachalol** 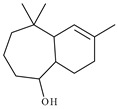	Antimicrobial	- *Aspergillus fumigatus* (MIC of 250 pg/mL and 46.4 pg/mL by the macro and microbroth dilution techniques, respectively)	[40]
	Antiparasitic	No reports were found	-
	Antiproliferative	- B16 F-10: murine melanoma cells (IC_50_ = 8.8 μg/mL and 7.3 μg/mL at 24 and 48 h, respectively)	[41]
**β-Pinene** 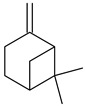	Antimicrobial	- *Candida albicans* (MIC from 117 to 6250 µg/mL)	[42]
	Antiparasitic	- *Trypanosoma cruzi* (% growth inhibition 95.7 ± 0.4 at 100 µg/mL, 89.9 ± 9.6 at 50 µg/mL, 71.6 ± 1.6 at 10 µg/mL and 12.3 ± 0.3 at 5 µg/mL)	[43]
	Antiproliferative	- Oral squamous cell carcinoma (IC_50_~67 µg/mL)	[44]

## Data Availability

All data are available in the article and the Supplementary Material.

## Supplementary Materials

The following supporting information can be downloaded at: https://www.mdpi.com/article/10.3390/molecules27061926/s1, Table S1: Chemical composition of the essential oils from *Baccharis parvidentata* (EO-Bp) and *Lippia origanoides* (EO-Lo), growing in Brazil.

## Sample Availability

There are no samples available.
