# Supplementary material for: In Vitro Pharmacological Screening of Essential Oils from Baccharis parvidentata and Lippia origanoides Growing in Brazil
Source: Molecules. 2022 Mar 16;27(6):1926. doi: 10.3390/molecules27061926 (PMC8953750; doi:10.3390/molecules27061926)
Supplement: Supplementary file 1 [file molecules-27-01926-s001.zip › Supplemental Table S1.pdf]

Table S1. Chemical composition of the essential oils from *Baccharis parvidentata* (EO-Bp) and *Lippia origanoides* (EO-Lo), growing in Brazil.

Compound	RI $_{calc}$	RI $_{lit}$	GC peak area (%)	
			B. parvidentata	*L. origanoides*
α-thujene	929	924	0.8	-
α-pinene	936	932	3.5	1.1
camphene	951	946	0.1	0.2
**sabinene**	975	969	**15.2**	0.1
**β-pinene**	978	974	**9.2**	0.4
myrcene	991	988	2.1	-
α-phellandrene	1005	1002	0.2	-
**δ-3-carene**	1012	1008	**5.7**	-
α-terpinene	1018	1014	1.2	-
o-cymene	1023	1020	0.2	-
p-cymene	1026	1022	0.1	-
**limonene**	1030	1024	4.1	0.5
cis-β-ocimene	1039	1032	0.2	0.1
trans-β-ocimene	1049	1044	0.4	0.5
ɣ-terpinene	1060	1054	2.0	0.1
terpinolene	1088	1086	0.6	-
linalool	1099	1095	1.1	0.2
borneol	1166	1165	-	0.1
terpinen-4-ol	1177	1174	4.8	0.2
α -terpineol	1190	1186	0.4	-
(*Z*)-methyl cinnamate	1303	1299	-	1.7
α-copaene	1373	1374	-	0.6
**(*E*)-methyl cinnamate**	1382	1376	-	**40.0**
β-elemene	1389	1389	0.1	-
***trans*-caryophyllene**	1415	1417	0.5	4.2
guaiene	1435	1437	-	0.2
α-himalechene	1444	1449	2.4	-
α-neoclovene	1449	1452	0.1	-
α-humulene	1449	1452	-	1.0
allo-aromandendrene	1456	1458	-	0.3
α-acoradiene	1467	1464	0.3	-
β-chamigrene	1473	1476	2.4	-
trans-cadina-1(6),4-diene	1473	1475	-	0.3
**germacrene D**	1477	1480	3.1	2.5
ɣ-himalechene	1479	1481	0.8	-
widdra-2,4(14)-diene	1482	1481	0.5	-
epi-cubebol	1492	1493	0.8	-
bicyclogermacrene	1495	1500	3.0	2.5
**α-muurolene**	1495	1500	-	0.3
β -himachalene	1499	1500	0.3	-
δ-cadinene	1519	1522	1.0	0.8
kessane	1522	1529	0.8	-
liguloxide	1524	1534	1.1	-
**hedycaryol**	1546	1546	1.9	**8.0**
longipinanol	1570	1567	0.8	-
spathulenol	1572	1577	-	0.7
guaiol	1600	1600	0.3	1.4
cubenol (1,10-di-epi)	1620	1618	3.4	-
eremoligenol	1624	1629	-	2.8
ɣ-eudesmol	1626	1630	0.8	3.5
epi-α-muurolol	1637	1640	0.8	-

hinesol	1637	1640	-	1.0
**himachalol**	1642	1652	**10.3**	-
**β-eudesmol**	1645	1649	-	**7.3**
α-cadinol	1647	1652	0.5	-
**α-eudesmol**	1649	1652	-	**7.6**
valerianol	1656	1656	0.8	0.3
7-epi-α-eudesmol	1662	1662	1.1	-
Total (%)			**90**	**91**

(-) no detection; RI_{calc}: linear retention index calculated; RI_{lit}: linear retention index reported in literature. In bold font are highlighted compounds with content higher than 5%. The oils were analyzed in an Agilent 7890A gas chromatograph equipped with mass spectrometry as described in Perera et al [9].
